# Phylogenetic Analysis and Characterization of a Sporadic Isolate of Equine Influenza A H3N8 from an Unvaccinated Horse in 2015

**DOI:** 10.3390/v10010031

**Published:** 2018-01-11

**Authors:** Chithra C. Sreenivasan, Sunayana S. Jandhyala, Sisi Luo, Ben M. Hause, Milton Thomas, David E. B. Knudsen, Pamela Leslie-Steen, Travis Clement, Stephanie E. Reedy, Thomas M. Chambers, Jane Christopher-Hennings, Eric Nelson, Dan Wang, Radhey S. Kaushik, Feng Li

**Affiliations:** 1 Department of Biology and Microbiology, South Dakota State University, Brookings, SD 57007, USA; chithra.sreenivasan@sdstate.edu (C.C.S.); sunayana.shyamjandhyala@sdstate.edu (S.S.J.); sisi.luo@jacks.sdstate.edu (S.L.); dan.wang@sdstate.edu (D.W.); radhey.kaushik@sdstate.edu (R.S.K.); 2 Cambridge Technologies, Oxford Street Worthington, MN 56187, USA; bhause@cambridgetechnologies.com; 3 Department of Veterinary and Biomedical Sciences, South Dakota State University, Brookings, SD 57007, USA; milton.thomas@sdstate.edu (M.T.); david.knudsen@sdstate.edu (D.E.B.K.); cpsteen@icloud.com (P.L.-S.); travis.clement@sdstate.edu (T.C.); jane.hennings@sdstate.edu (J.C.-H.); eric.nelson@sdstate.edu (E.N.); 4 Gluck Equine Research Center, University of Kentucky, Lexington, KY 40546, USA; sereed0@uky.edu (S.E.R.); tmcham1@uky.edu (T.M.C.); 5 BioSNTR, Brookings, SD 57007, USA

**Keywords:** equine influenza H3N8, horses, Florida Clade 1, lineages, hemagglutinin

## Abstract

Equine influenza, caused by the H3N8 subtype, is a highly contagious respiratory disease affecting equid populations worldwide and has led to serious epidemics and transboundary pandemics. This study describes the phylogenetic characterization and replication kinetics of recently-isolated H3N8 virus from a nasal swab obtained from a sporadic case of natural infection in an unvaccinated horse from Montana, USA. The nasal swab tested positive for equine influenza by Real-Time Quantitative Reverse Transcription Polymerase Chain Reaction (RT-PCR). Further, the whole genome sequencing of the virus confirmed that it was the H3N8 subtype and was designated as A/equine/Montana/9564-1/2015 (H3N8). A BLASTn search revealed that the polymerase basic protein 1 (PB1), polymerase acidic (PA), hemagglutinin (HA), nucleoprotein (NP), and matrix (M) segments of this H3N8 isolate shared the highest percentage identity to A/equine/Tennessee/29A/2014 (H3N8) and the polymerase basic protein 2 (PB2), neuraminidase (NA), and non-structural protein (NS) segments to A/equine/Malaysia/M201/2015 (H3N8). Phylogenetic characterization of individual gene segments, using currently available H3N8 viral genomes, of both equine and canine origin, further established that A/equine/Montana/9564-1/2015 belonged to the Florida Clade 1 viruses. Interestingly, replication kinetics of this H3N8 virus, using airway derived primary cells from multiple species, such as equine, swine, bovine, and human lung epithelial cells, demonstrated appreciable titers, when compared to Madin–Darby canine kidney epithelial cells. These findings indicate the broad host spectrum of this virus isolate and suggest the potential for cross-species transmissibility.

## 1. Introduction

Equine influenza epizootics, which affect horses, zebra, mules, and donkeys all over the world, are characterized by an acute dry cough, high body temperature, mucopurulent nasal discharge, lethargy and anorexia [1,2,3,4,5]. Vaccination failure, the mobility of unvaccinated horses and insufficient quarantine measures are major predisposing factors for equine influenza occurrence in places where EIV is endemic [6]. Equine influenza viruses are type A viruses and the major subtypes affecting horses include H7N7 and H3N8 which were previously designated as equine 1 and equine 2 viruses. However, currently, H3N8 [7] is the only subtype affecting worldwide equine populations. A1/equine/Prague/56 was the first H7N7 prototype isolated from Prague, Czech Republic, which affected horses in Sweden and Eastern Europe around 1955–1956 [8,9,10,11]. Equine H3N8 prototype, A2-equine/Miami/63 was originally isolated from Miami, Florida in 1963, as an upper respiratory disease manifestation in imported animals from Argentina [8,12,13,14]. Since 1979, only H3N8 variants have been found in circulation, even though both H7N7 and H3N8 co-circulated in the equine populations in earlier times [6,11,15]. It is believed that homo/intra and hetero/inter-subtype reassortments have played a crucial role in the evolution dynamics of equine influenza virus (EIV), where intra-subtype reassortments enhanced virulence in EIV H3N8, and inter-subtype reassortments led to the extinction of EIV H7N7 [16]. Compared to H7N7, H3N8 affects horses of all ages, irrespective of vaccination coverage. Previous studies on the phylogeny of EIV from different parts of the world have clearly demonstrated the evolution of equine influenza viruses, particularly the H3N8 subtype, which is the prominent subtype in circulation [7,17,18,19,20,21]. EIV H3N8 has been causing major transcontinental pandemics across the world and has crossed over species to successfully establish in the canine host since 2004 [22].

The equine H3N8 subtype diverged genetically into American and Eurasian lineages around the 1980s, however American lineages have been circulating in Europe and vice-versa [15,23]. No cases of Eurasian lineages have been reported since 1994 [16]. Around 1990, the American lineage diverged antigenically and genetically into Kentucky, South America and Florida sub-lineages with the Florida sub-lineage circulating predominantly [1]. Around the early 2000s, the Florida sub-lineage diverged into two clades: Clade 1 (FC1) and Clade 2 (FC2) [23,24,25]. Even though the clade separation was not so pronounced for the H3N8 isolates in 2002–2003, the divergence became indisputable for strains isolated after 2005 [26]. Since 2007, all Asian and European isolates have been found to belong to FC2, whereas North American isolates belong to FC1, but the reverse can also occur, owing to the increased international mobility of the animals for races and exhibition. Such activities can lead to loose transmission bottle-neck and mixed infections, which may contribute to vaccination failures [16,27].

Cross-species transmission of EIV in canines, characterized by the complete genome transfer of equine H3N8, occurred initially in the United States, followed by the UK and Australia in the early 2000s [28,29]. Canine influenza, caused by H3N8, has been associated with fever, cough, suppurative pneumonia and per-acute death [28,30,31,32]. Both cats and calves have been experimentally infected with EIVs and the infected animals showed clinical symptoms, virus shedding and demonstrated contact transmission [33,34,35]. Interestingly, two H3N8 strains of equine origin, with close relation to the European H3N8 EIVs, were isolated from pigs in China during 2004–2006 [35]. Experimental infection of humans with EIV H3N8 was demonstrated more than 50 years ago by the National Institute of Health and it was found that humans are susceptible to H3N8 with clinical manifestations [36,37,38]. Further, serological evidence of EIV H3N8 has been reported in humans with occupational exposure indicating its zoonotic potential [39]. These evidence of cross-species transmission and broad host spectrum raise the possibility of zoonosis and thus could have a serious implication on public health.

Previous outbreaks in vaccinated and unvaccinated horses in Europe in 1979 and 1989 showed that EIV can undergo a rapid rate of antigenic drift and cause vaccination failures [40,41]. The Office International des Epizooties (OIE) vaccine recommendations remain unchanged since 2010, and mandates to include both clades—FC1 represented by A/eq/South Africa/04/2003-like or A/eq/Ohio/2003-like viruses and FC2 represented by A/eq/Richmond/1/2007-like viruses. The OIE also recommends periodical update of the vaccine strains, based on the epidemiological survey results [42,43]. While, vaccination is the only solution to effectively control and prevent EIV outbreaks, the continuous antigenic drift between the strains poses a serious threat for necessary protection, especially for horses involved in cross country events [43,44]. Continued surveillance and reporting of EIV from different countries is of utmost importance, to ensure the effective coverage of circulating strains by the current vaccine strains and thereby boosts herd immunity.

The aim of this study was to characterize an equine H3N8 virus isolate, obtained from a 3-year-old unvaccinated gelding showing respiratory disease, from Montana, USA, in 2015. Phylogenetically distinct clades/lineages of EIV have been co-circulating globally, undergoing gene reassortments, thereby posing a serious challenge in the selection of vaccine strains. Hence, understanding the evolutionary profile of EIV is imperative to estimate the phylogenetic diversity and distribution of equine influenza viruses. Here, we describe the phylogenetic characterization of all the eight gene segments of this recently isolated EIV. Our phylogenetic analysis inferred that the polymerase basic protein 1 (PB1), polymerase acidic (PA), and nucleoprotein (NP) segments of A/equine/Montana/9564-1/2015 clustered with A/equine/Tennessee/29A/2014 and hemagglutinin (HA), polymerase basic protein 2 (PB2), neuraminidase (NA), matrix (M), and non-structural (NS) segments clustered with A/equine/Malaysia/M201/2015 and A/equine/Tennessee/29A/2014, both belong to Clade 1 (FC1) viruses of Florida sub-lineage [45]. To test the in vitro cross-species susceptibility and zoonotic potential, we infected A/equine/Montana/9564-1/2015 on airway derived primary cells from different mammalian species such as swine, bovine, and equine, along with human lung epithelial A549 cells and MDCK cells from canine origin. Our results showed that A/equine/ Montana/9564-1/2015 productively replicated in these cell lines, suggestive of its broad host spectrum and possible cross-species transmissibility.

## 2. Materials and Methods

### 2.1. Case Description

In May 2015, nasal swabs were sent to the Animal Disease and Research Diagnostic Laboratory (ADRDL), South Dakota State University from three horses showing respiratory disease from Montana, USA. Among the three horses, only one tested positive for equine influenza A by Real-Time Quantitative Reverse Transcription Polymerase Chain Reaction (qRT-PCR) and one of the other two animals tested positive for *Streptococcus equi*. The influenza A positive horse was an unvaccinated 3-year-old gelding and the case was reported as a sporadic case of natural infection. The virus was isolated by passaging on MDCK cells and designated as A/equine/Montana/9564-1/2015.

### 2.2. Whole Genome Sequencing and Phylogenetic Analysis

Whole genome sequencing of A/equine/Montana/9564-1/2015 propagated on MDCK cells was performed using the Illumina MiSeq instrument and Nextera XT library preparation kit (San Diego, CA, USA), as described previously [46]. Whole genome sequencing confirmed it as a case of equine influenza A, H3N8 subtype. The sequences were submitted to the NCBI GenBank (Accession #s MG198996-MG199003). A BLASTn search analysis, optimized for highly similar sequences (megablast), was conducted for all the eight gene segments of A/equine/Montana/9564-1/2015 (H3N8) [47].

Both canine and equine nucleotide sequences of H3N8 subtype were acquired from the influenza virus resource (https://www.ncbi.nlm.nih.gov/genomes/FLU/Database/nph-select.cgi?go=database, accessed 5 January 2018) and phylogenetic analyses were performed using MEGA 7.0 [45,48]. Nucleotide sequences were aligned using MUltiple Sequence Comparison by Log-Expectation (MUSCLE) and the evolutionary history of each segment was inferred by constructing maximum likelihood trees, using the best nucleotide substitution models, suggested by the ‘test for best DNA/ protein fitness’ in MEGA 7.0 [48,49]. The best nucleotide substitution models inferred for maximum likelihood trees for the gene segments were general time-reversible with gamma distributed with invariant sites (GTR+G+I) for *PB2*, *PB1*, *NP*; Tamurai–Nei with gamma distributed with invariant sites (TN93+G+I) for *PA*, *HA*; general time-reversible with gamma distributed (GTR+G) for *NA*; Hasegawa-Kishino-Yano with gamma distributed with invariant sites (HKY+G+I) for *M*; Hasegawa–Kishino–Yano with gamma distributed (HKY+G) for *NS* [50,51,52]. All nucleotide positions containing gaps and missing data were partially deleted and very strong branch filters were applied to run the analysis. For each taxon, the bootstrap value was determined from 1000 replicates to verify the tree topology.

### 2.3. Virus and Cell Culture

A/equine/Montana/9564-1/2015 virus, isolated from a nasal swab and propagated on MDCK cells, was a generous gift from the ADRDL. MDCK cells, maintained in Dulbecco’s Modified Eagle medium, supplemented with 10% fetal bovine serum (FBS) (PAA Laboratories Inc., Dartmouth, MA, USA) and penicillin-streptomycin (Life Technologies, Carlsbad, CA, USA) (100 U/mL) were used in this study. MDCK cells, cultured in the T-75 flask, were inoculated with the virus inoculum at 0.01 multiplicity of infection (MOI) and incubated at 37 °C in 5% CO_2_ for 1 h. Following infection, the virus growth medium, consisting of fresh DMEM with 0.3% bovine serum albumin (BSA), 1 μg/mL tolylsulfonyl phenylalanyl chloromethyl ketone (TPCK)-treated trypsin (Sigma, Saint Louis, MO, USA) and penicillin-streptomycin (100 U/mL) (Life Technologies, Carlsbad, CA, USA), was added for further incubation at 37 °C in 5% CO_2_ for 48–72 h. The infected cell cultures were freeze-thawed. The supernatant was spun at 500× *g* for 10 min at 4 °C to remove the cellular debris. Determination of virus titers in MDCK cells was done according to the Reed and Muench method [53].

### 2.4. Replication Kinetics

Replication kinetics of A/equine /Montana/9564-1/2015 were studied using airway primary cells, derived from different mammalian species, such as equine primary tracheal myofibroblasts (EPTrF), swine tracheal and lung primary epithelial cells (SPTrE, SPLE), bovine primary nasal turbinate cells (BPT) along with the human lung carcinoma cell line, A549 and MDCK. The primary cells were grown in Dulbecco’s Modified Eagle Medium (DMEM/F-12), 5% FBS (PAA Laboratories Inc., Dartmouth, MA, USA) and penicillin-streptomycin (100 U/mL) (Life Technologies, Carlsbad, CA, USA), Mouse epidermal growth factor (EGF) (Sigma (5 ng/mL)), insulin (5 µg/mL) transferrin (5 µg/mL) and selenium (5 ng/mL) (Sigma). A/equine/Montana/9564-1/2015 was used at 1 MOI for infection of the primary cells and A549 and 0.01 MOI for infection of MDCK cells. After the infection, samples were collected at 12 h intervals for 72 h. The samples were titrated on MDCK cells to find the 50% tissue culture infective dose (TCID_50_/mL) as per the Reed and Muench formula.

## 3. Results

### 3.1. BLAST Analysis

Whole genome sequencing was performed, and the virus was identified as EIV subtype H3N8 and designated as A/equine/Montana/9564-1/2015 (H3N8). The sequences of the eight segments—polymerase basic protein 2 (PB2), polymerase basic protein 1 (PB1), polymerase acidic (PA), hemagglutinin (HA), nucleoprotein (NP), neuraminidase (NA), matrix (M1 and CM2), and non-structural proteins (NS1, NS2)—were obtained in full length, and sizes were 2341, 2274, 2151, 1762, 1572, 1410, 982, and 838 nucleotides (nt) respectively. Only the NA segment was nearly complete, with 20 and 30 nt missing at the 5′ and 3′ terminal regions, respectively. The sequences were submitted to GenBank and accession numbers were assigned: MG198996 (*PB2*), MG198997 (*PB1*), MG198998 (*PA*), MG198999 (*HA*), MG199000 (*NP*), MG199001 (*NA*), MG199003 (*M*), and MG199003 (*NS*). The BLASTn search analysis, optimized for highly similar sequences (megablast) for all the eight segments of A/equine/Montana/9564-1/2015, demonstrated the highest percentage identity to the H3N8 strains of equine origin and not to any of the H3N8 strains of canine origin, indicating the absence of any species cross over and intra-subtype genetic exchange.

A/equine/Montana/9564-1/2015 demonstrated equally high percentage identity to six H3N8 strains isolated from Tennessee in 2014 (A/equine/Tennessee/4A/2014, A/equine/Tennessee/27A/2014, A/equine/Tennessee/28A/2014, A/equine/Tennessee/28B/2014, A/equine/Tennessee/29A/2014, A/equine/Tennessee/30A/2014) and A/equine/Malaysia/M201/2015. Considering the sequence similarity between these highly homologous strains, we used A/equine/Tennessee/29A/2014 as a representative strain for BLASTn results and phylogenetic analysis. A/equine/Montana/9564-1/2015 showed a percent identity score of 99.78 (*PB1*), 99.81 (*PA*), 99.54 (*HA*), 99.87 (*NP*), and 99.8 (*M*) to A/equine/Tennessee/29A/2014, while the *PB2*, *NA* and *NS* segments demonstrated percent identities of 99.83, 99.93 and 100 respectively, to A/equine/Malaysia/M201/2015 (Table 1). The *HA* segment of A/equine/Montana/9564-1/2015 also showed percent identity scores of 99.23% and 99.18% with two strains from Malaysia, A/equine/Malaysia/M201-2/2015 and A/equine/Malaysia/M201-1/2015. Overall, A/equine/Montana/9564-1/2015 shared the highest percent identity with two equine H3N8 viruses of Florida Clade 1 sublineage, A/equine/Tennessee/29A/2014 and A/equine/Malaysia/M201/2015.

### 3.2. Phylogenetic Analysis

To estimate the evolutionary history of A/equine/Montana/9564-1/2015, we performed phylogenetic analyses of all the eight gene segments, with the currently available sequences of both canine and equine H3N8 subtypes in the influenza virus resource database [45,54]. The EIV sequences we analyzed, included the sequences from pre-divergence, Eurasian and American lineages (Kentucky and South American sub-lineages) and Clades 1 and 2 of the Florida sub-lineage. The total number of canine and equine sequences of H3N8 subtype, used to construct the phylogenetic trees for each segment were *PB2* (124), *PB1* (130), *PA* (120), *HA* (161), *NP* (122), *NA* (161), *M* (121) and *NS* (121). For the phylogenetic analyses, we used a fixed number of nucleotides for each gene segment: *PB2* (2280), *PB1* (2274), *PA* (2151), *HA* (1695), *NP* (1497), *NA* (1410), *M* (982), and *NS* (838).

**Figure 1:**
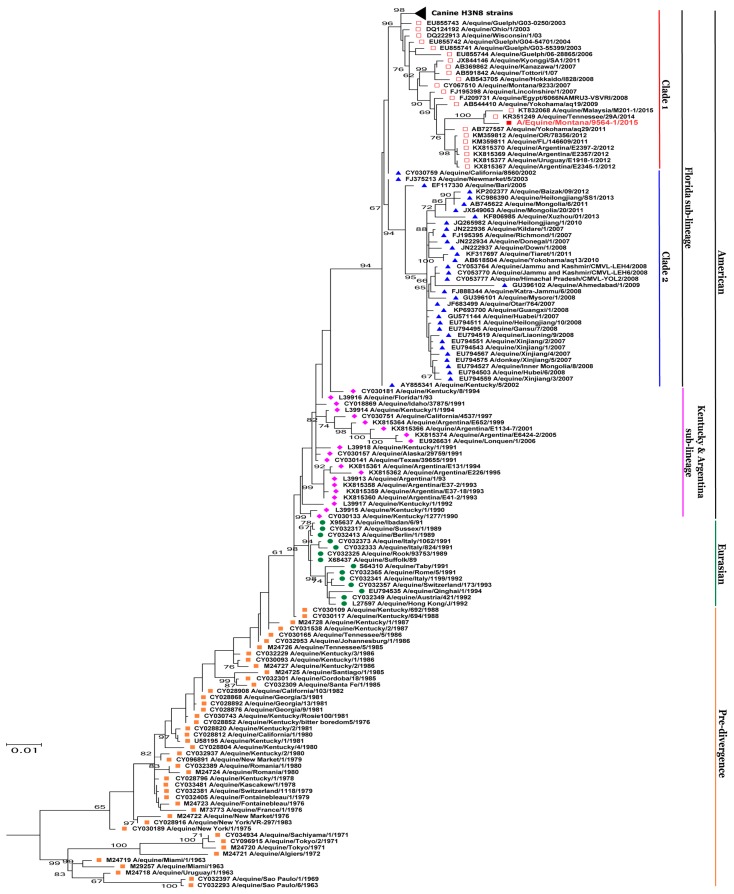
Phylogenetic analysis of the *HA* sequence. The evolutionary history of hemagglutinin (HA) nucleotide sequence of A/equine/Montana/9564-1/2015 was inferred using the maximum likelihood method by MEGA 7.0 [48], with a bootstrapping of 1000 replicates. The analysis involved 161 sequences of both canine and equine origin, and canine H3N8 strains were shown as a collapsed branch. Different phylogenetic groups of equine influenza virus (EIV) were color-coded and marked. Orange filled square = pre-divergence; Green filled circle = Eurasian; pink filled diamond = American lineage (Kentucky + Argentina); Blue filled triangle = Florida sub-lineage Clade 2; Red Open square = Florida sub-lineage Clade 1. Bootstrap values are shown at each node and A/equine/Montana/9564-1/2015 is highlighted in red.

**Figure 2:**
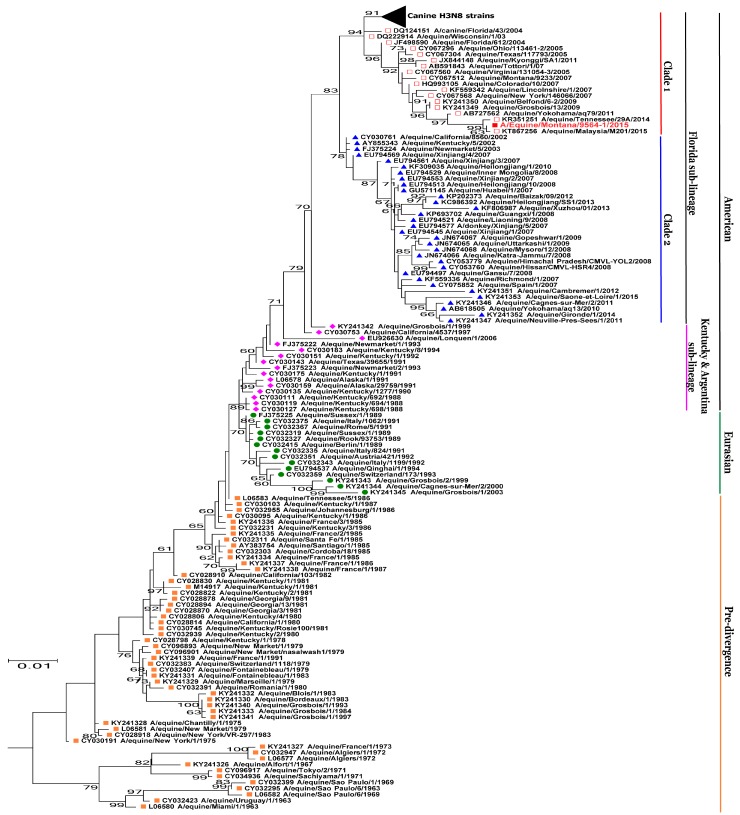
Phylogenetic analysis of the *NA* sequence. The evolutionary history of neuraminidase (NA) nucleotide sequences of A/equine/Montana/9564-1/2015 was inferred using the maximum likelihood method by MEGA 7.0 [48], with a bootstrapping of 1000 replicates. The analysis involved 161 sequences of both canine and equine origin, and canine H3N8 strains were shown as a collapsed branch. Different phylogenetic groups of EIV were color-coded and marked. Orange filled square = pre-divergence; Green filled circle = Eurasian; pink filled diamond = American lineage (Kentucky + Argentina); Blue filled triangle = Florida sub-lineage Clade 2; Red Open square = Florida sub-lineage Clade 1. Bootstrap values are shown at each node and A/equine/Montana/9564-1/2015 is highlighted in red.

The evolutionary history of the eight viral gene segments obtained by the phylogenetic analyses was in complete agreement with the percent identity score obtained by BLASTn, clustering with equine influenza A H3N8 viruses and not with canine H3N8 viruses. The evolutionary history of *HA* and *NA* segments of A/equine/Montana/9564-1/2015, was shown as complete trees, in which the different phylogenetic groups of EIV were color-coded and grouped to describe the pre-divergence, Eurasian and American lineages (strains from Kentucky and Argentina/South American sub-lineages), the Florida sub-lineage and Clade 1 and Clade 2 viruses (Figure 1 and Figure 2). The phylogenetic tree of the *NA* segment of our isolate, clustered with A/equine/Malaysia/M201/2015 and A/equine/Tennessee/29A/2014 (Figure 2). A similar cluster can be seen in the phylogenetic tree of *HA*. The *HA* segment of A/equine/Malaysia/M201/2015 was not available in the database; however, the *HA* segment of A/equine/Montana/9564-1/2015 clustered with another strain from Malaysia, i.e., A/equine/Malaysia/M201-1/2015 along with A/equine/Tennessee/29A/2014 (Figure 1). The oldest H3N8 EIV isolates, dated from 1963 to 1988, were grouped under the pre-divergence lineage and the isolates from Europe and Asia from 1989 to 1994 were grouped under the Eurasian lineage [18]. Isolates, dated from 1990 to 2001, that belonged to the Kentucky and Argentina/South American sub-lineages, were grouped under American lineage, along with Florida sub-lineage Clade 1 and Clade 2 viruses, isolated from 2005 to present [23,26,55].

**Figure 3:**
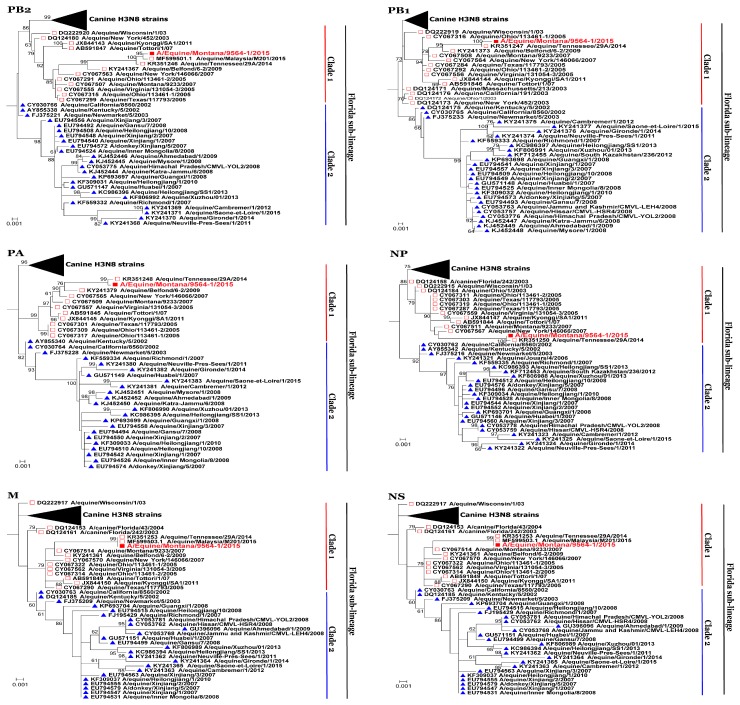
Phylogenetic trees of *PB2*, *PB1*, *NP*, *NA*, *M* and *NS* sequences. Phylogenetical analyses of nucleotide sequences of *PB2*, *PB1*, *PA*, *NP*, *M*, and *NS* segments of A/equine/Montana/9564-1/2015 were inferred using the maximum likelihood method by MEGA 7.0 [48], with a bootstrapping of 1000 replicates. The analysis involved EIV sequences of both canine and equine origin, and canine H3N8 strains were shown as a collapsed branch. Subtrees, involving Florida sub-lineage Clade 1 (Red open square) and Florida Clade 2 (Blue filled triangle) viruses, are shown. Bootstrap values are shown at each node and A/equine/Montana/9564-1/2015 is highlighted in red.

Phylogenetic analyses of all the other six segments (*PB2*, *PB1*, *PA*, *NP*, *M*, *NS*) of A/equine/Montana/9564-1/2015 were performed. The *PB1*, *PA*, and *NP* segments clustered with A/equine/Tennessee/29A/2014 while *PB2, M* and *NS* segments clustered with A/equine/Malaysia/M201/2015 and A/equine/Tennessee/29A/2014, both belonged to the Clade 1 equine influenza viruses of Florida sub-lineage, of American lineage (Figure 3). The phylogeny of these six segments has been represented by subtrees, involving only Florida Clade 1 and Florida Clade 2 sub-lineages of American lineage (Figure 3).

### 3.3. Viral Replication Kinetics

**Figure 4:**
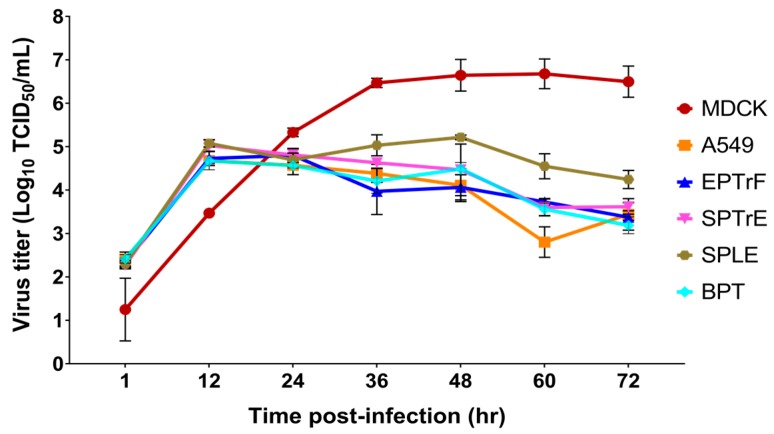
Replication kinetics of A/equine/Montana/9564-1/2015 in swine primary tracheal epithelial cells (SPTrE), swine primary lung epithelial cells (SPLE), bovine primary turbinate cells (BPT), equine primary tracheal myofibroblasts (EPTrF), human lung epithelial cell line (A549) and MDCK cells. Note that 0.01 multiplicity of infection (MOI) was used for MDCK infection, while 1.0 MOI was used for infection of other cell types. The samples were taken at 12 h intervals during a 72 h period and viral 50% infective doses were calculated to determine log_10_ TCID_50_/mL. The values are shown as mean ± SEM and plotted as a function of time.

A/equine/Montana/9564-1/2015 was propagated in MDCK cells and the infectivity of the virus was determined by hemagglutination assay (HA) and by 50% tissue culture infective dose (TCID_50_/mL). To explore the in vitro cross-species susceptibility of this virus isolate, we used a panel of six cells, which included the airway primary cells derived from equine, swine and bovine species and A549 from human lung epithelium to determine the infectivity of A/equine /Montana/9564-1/2015. An MOI of 1.0 was used for infection of these cell types because lower MOIs failed to produce measurable replication kinetics. For MDCK cells, a cell line routinely used to replicate equine influenza viruses, only 0.01 MOI of the virus was used for infection. Interestingly, A/equine/Montana/9564-1/2015 productively replicated in swine primary tracheal epithelial cells (SPTrE), swine primary lung epithelial cells (SPLE) and bovine primary nasal turbinate (BPT), yielding peak titers of 5.03 (12 hpi), 5.21 (48 hpi) and 4.67 log_10_ TCID_50_/mL (12 hpi), respectively (Figure 4). Equine primary tracheal myofibroblasts also yielded a comparable peak titer of 4.8 log_10_ TCID_50_/mL at 24 hpi, whereas A549, the human lung epithelial cell line, supported A/equine/Montana/9564-1/2015 with a peak titer of 4.68 logs at 12 hpi. Infected MDCK cells demonstrated a peak titer of 6.8 log_10_ TCID_50_/mL, which was 2 logs higher than those seen in primary cells, in spite of the low MOI used for infection. Overall, A/equine/Montana/9564-1/2015 productively replicated in all the six types of cells derived from swine, bovine, equine, human and canine to very appreciable titers (Figure 4).

## 4. Discussion

EIV H3N8 epidemics have been reported worldwide, on a large scale, since the first reported case of H3N8 occurred in Florida in 1963. Just like any other influenza epidemic in the past, EIV epidemics occur in vaccinated and immunologically naïve populations, in particular, H3N8 EIV epidemics can happen in horses of all ages, regardless of vaccination status [7,56,57,58]. Vaccination breakdowns have been associated with EIV outbreaks in the past, as documented in thoroughbred yearlings in Kentucky, and several other parts of the world, including France and Ireland and horses imported into South Arabia and Japan [58]. The report we presented here was a sporadic case, which occurred in an unvaccinated 3-year-old gelding, characterized by clinical manifestations. The two other horses from the ranch exhibiting respiratory symptoms tested negative for influenza by qRT-PCR. As such, we concluded that this could be a sporadic infection. There is no information available pertaining to the movement or involvement of any social events/shows. Such cases of sporadic infection usually occur due to the mobility of inadequately quarantined, or sub-clinically infected, vaccinated or unvaccinated horses, into unvaccinated or inadequately vaccinated herds with little or no immunity [16,32].

According to the OIE Expert Surveillance Panel on Equine Influenza Vaccine Composition, the year 2015 witnessed an increased activity in H3N8 EIV cases, reported from 46 premises over 23 states in the USA [43]. In 2016, 30 confirmed EIV cases were reported from 16 states [43]. Unfortunately, there is no vaccination data available on these outbreaks from the USA. In 2015–2016, EIV outbreaks were reported in other parts of the world, such as Ireland, Sweden, and the UK. The outbreaks reported in the UK occurred in unvaccinated animals. As per the report, no cases have been reported from Asia and South America during this period [43].The OIE report also concluded that EIV H3N8 viruses isolated from the USA in 2016 were homologous to the isolates from 2015 and belonged to the Florida sub-lineage Clade 1 (FC1). On the other hand, viruses detected from the UK in 2015–2016 were Florida sub-lineage Clade 2 (FC2) viruses. The data we obtained from phylogenetic analysis were in complete agreement with this observation.

The BLASTn analysis of all viral genome segments, except *PB2*, *NA*, and *NS*, showed the highest percentage identity to six H3N8 EIV strains isolated from Tennessee in 2014. Considering the percentage identity between these six highly homologous strains, we used A/equine/Tennessee/29A/2014 as a representative strain for our phylogenetic analysis. It was very difficult to conclude that this sporadic infection occurred from the contaminated premises, as there were no other horses on the premises reported to have EIV infection, according to available information to us. We also checked the influenza virus database (https://www.ncbi.nlm.nih.gov/genomes/FLU/Database/nph-select.cgi?go=database, accessed 5 January 2018) to gather information on the EIV isolates reported from North America from 2000 to 2017. It is noteworthy that, only 1/69 isolates (A/equine/Montana/9233/2007(H3N8)) have been reported from Montana during the past 17 years. According to the report from the OIE Expert Surveillance Panel, EIV cases occurred in the USA in 2015–2016 [43]. However, we could not find any documentation of new recent EIV isolates from North America during 2015–2017, which indicates either the new isolates are homologous to the old known strains or lack of reporting of the new isolates. The phylogenetic analysis, using the maximum likelihood algorithm, used all the available full-length sequences of H3N8 currently available in the database, of both equine and canine origins. The phylogenetic reconstruction of the individual genes was in complete agreement with the BLAST results and A/equine/Montana/9564-1/2015 clustered with FC1 viruses, originated in the USA. The fact that *PB2*, *NA*, *M*, *NS* and *HA* segments clustered with A/equine/Malaysia/M201/2015 (H3N8) is interesting, because Malaysia has been EIV free since 1977 and this outbreak in 2015, which started as a sporadic case occurred in a time frame, concurrent with the import of four racehorses (http://www.oie.int/wahis_2/public/wahid.php/Reviewreport/Review?reportid=19160, accessed 5 January 2018). Historically, EIV originating from Asia and Europe, usually clusters with Florida Clade 2 viruses. On the contrary, A/equine/Malaysia/M201/2015 clustered with Florida Clade 1 viruses, together with A/equine/Tennessee/29A/2014. It is difficult to trace the time and means of introduction of A/equine/Malaysia/M201/2015 in the USA. It is worth noting that the sequences currently available in the database for A/equine/Malaysia/M201/2015 are only for four segments, i.e., *PB2*, *NA*, *M*, and *NS*. Only *HA* sequence is available for A/equine/Malaysia/M201-1/2015. The BLASTn percent identity score of *M* segment showed 99.8% and 99.59% to A/equine/Tennessee/29A/2014 and A/equine/Malaysia/M201/2015, respectively. Had there been all eight segments available, A/equine /Montana/9564-1/2015 might have shown a higher sequence identity to A/equine/Malaysia/M201/2015 than Tennessee 2014 strains. Overall, A/equine/Montana/9564-1/2015 viral segments clustered with Florida Clade 1 viruses, A/equine/Tennessee/29A/2014 and A/equine/Malaysia/M201/2015 and hence, belong to the Florida Clade 1 group of equine influenza viruses.

Equine H3N8 viruses are known to have jumped the species barrier and have successfully established infections in canines, causing canine influenza epidemics and epizootics. Experimental infection of EIV has been demonstrated in vitro [59,60] and in vivo in calves, and cats [33,34]. An in vitro study by Feng et al. also demonstrated robust infection in MDCK, fibroblast cells from the dog (A72) and Norden Laboratories feline kidney (NLFK), moderate infection in *Mustela putorius furo* (Mpf) cell line from ferrets and poor infection in A549 and equine kidney cells (EQKD) [61]. Further, two H3N8 viruses of equine origin have been isolated from pigs in China during 2004–2006 and these strains were found to be closely related to the European H3N8 strains from the early 1990s [35]. Previous studies have shown that pigs are the ‘mixing vessels’—harboring avian, swine and human influenza viruses—which facilitate gene reassortment, giving rise to novel reassortant influenza viruses with high transmissibility to humans [62]. Hence, the isolation of H3N8 viruses of equine origin from pigs in China demands attention, as the establishment and dissemination of the reassortant mammalian-adapted viruses can happen elsewhere in the world. The zoonotic potential of EIV has not been documented, even though there has been occasional serological evidence in occupational workers [39]. Nevertheless, EIV H3N8 has the potential to infect humans, as demonstrated in the experimental infection of human volunteers with EIV H3N8 and influenza-like symptoms were detected in this cohort [36,37,38]. In this study, we used species-specific primary respiratory epithelial cells, of swine, bovine and equine origin, to evaluate the replication competency of A/equine /Montana/9564-1/2015 and this was compared to MDCK, a cell line widely used for influenza studies [63]. The airway primary cells we used were non-transformed cells and can mimic the normal physiological environment. To represent respiratory cells from human origin, we used human lung epithelial cell line A549, which is yet another cell line used for influenza studies [64,65]. The increase in the viral titer demonstrated in the MDCK cells by 2 logs, at a lower MOI of 0.01, could be due to the adaptation of this EIV isolate to grow in MDCK cells, as the virus was initially isolated in MDCK cells [63]. Compared to MDCK cells, we used 100 times more MOI on non-transformed primary cells from bovine, swine and equine and also on the human lung epithelial cell line, A549, because lower MOIs failed to produce measurable replication kinetics. The explanation for the low virus titer in these primary cells and A549, despite using a high MOI, could be attributed to the differences in receptor preference or replication competence in different cell types and species-specific innate immune responses of the cells, posing a barrier to restrict the replication of this H3N8 virus [63,66]. Our data suggested the potential for cross-species susceptibility. However, these results should only be interpreted after taking into consideration the inherent limitations of translating in vitro findings to in vivo conditions.

Overall, this study provided us insights about the evolutionary relationship and in vitro cross-species infectivity of A/equine /Montana/9564-1/2015 (H3N8) virus. A comprehensive genome-scale analysis of new isolates is essential to understand the molecular evolution and phylodynamics of EIV, which in turn would help in the strategic selection of vaccine strains, effective surveillance, and control. Antigenic and genetic variations caused by evolutionary processes play a critical role in determining the dynamics of host range and tropism of influenza viruses. Further in vivo studies are needed to evaluate the cross-species transmissibility of EIV H3N8 and its ability to cause infections and respiratory diseases in other mammalian hosts, including humans.

## Figures and Tables

**Table 1:** Equine influenza viruses with the highest nucleotide identity to A/equine/Montana/9564-1/2015, determined by the BLASTn.

Gene	Accession No.	Viruses with Highest % of Nucleotide Identity	Percent Identity
*PB2*	MF599501.1	A/equine/Malaysia/M201/2015 (H3N8)	99.83
*PB1*	KR351247.1	A/equine/Tennessee/29A/2014 (H3N8)	99.78
*PA*	KR351248.1	A/equine/Tennessee/29A/2014 (H3N8)	99.81
*HA*	KR351249.1	A/equine/Tennessee/29A/2014 (H3N8)	99.54
*NP*	KR351250.1	A/equine/Tennessee/29A/2014 (H3N8)	99.87
*NA*	KT867256.1	A/equine/Malaysia/M201/2015 (H3N8)	99.93
*M*	KR351252.1	A/equine/Tennessee/29A/2014 (H3N8)	99.8
*NS*	MF599503.1	A/equine/Malaysia/M201/2015 (H3N8)	100.0

Polymerase basic protein 2 (*PB2*), Polymerase basic 1 (*PB1*), Polymerase acidic (*PA*), Hemagglutinin (*HA*), Nucleoprotein (*NP*), Neuraminidase (*NA*), Matrix (*M*), Non-structural protein (*NS*).
